# Cutaneous leishmaniasis: epidemiology, treatment access and translational challenges of topical therapies

**DOI:** 10.3389/fmicb.2025.1588311

**Published:** 2026-01-12

**Authors:** Dolores C. Carrer, Francesca Papera, Diego N. Ríos

**Affiliations:** 1 Instituto de Investigación Médica Mercedes y Martín Ferreyra, INIMEC (CONICET, UNC), Córdoba, Argentina; 2 Facultad de Ciencias Exactas, Físicas y Naturales, Universidad Nacional de Córdoba, Córdoba, Argentina

**Keywords:** cutaneous leishmaniasis, availability, topical treatments, neglected tropical disease, clinical trials

## Abstract

Leishmaniasis is an orphan, vector-borne parasitic disease endemic in more than 90 countries. It displays different clinical manifestations, being the cutaneous form (CL) the most common. This presents as skin ulcers that produce significant psychosocial distress, lifelong scarring and stigmatization. Historically endemic in low-income regions in the tropics, epidemiological data and computational models forecast the continued expansion into regions further away from the Equator, both northwards and southwards. Treatments for CL are unsatisfactory and are currently the major unmet medical need for the leishmaniases. An inherent difficulty with using the available systemic drugs is that they are highly toxic and painful to administer and require second-level hospital infrastructure to manage side effects. In this context, local treatments, and in particular topical treatments for CL are particularly interesting due to their potential to be efficacious and less toxic, painful and inconvenient than systemic treatments. They could improve patient compliance and allow self-treatment, diminishing associated financial costs both for the patients and for the states that usually provide the treatments. In this work we discuss epidemiology of the disease and availability of treatments and then center on topical treatments, covering advances in preclinical and clinical studies.

## 1 Introduction

Leishmaniasis is a vector-borne parasitic, zoonotic disease caused by protozoa of the genus *Leishmania*. It is transmitted from a mammal reservoir to humans by a phlebotomine insect (sand fly), and displays different clinical manifestations, ranging from asymptomatic or subclinical infection to disfiguring forms of cutaneous (CL), mucocutaneous, (MCL) or potentially fatal visceral leishmaniasis (VL). The polymorphic outcome of *Leishmania* infection depends on the virulence of the infecting parasite strain, as well as the host’s immune response. CL is the most common form, presenting as skin lesions that may lead to lifelong scarring and stigmatization. Old World cutaneous leishmaniasis, seen in the Eastern hemisphere, is caused by *L. donovani, L. infantum, L. major, L. tropica and L. aethiopica*, while New World cutaneous leishmaniasis, which refers to American cases, is predominantly caused by *L. braziliensis, L. panamensis, L. guyanensis, L. amazonensis, L. mexicana and L. peruviana* (Madusanka et al., 2022).

**Figure 1:**
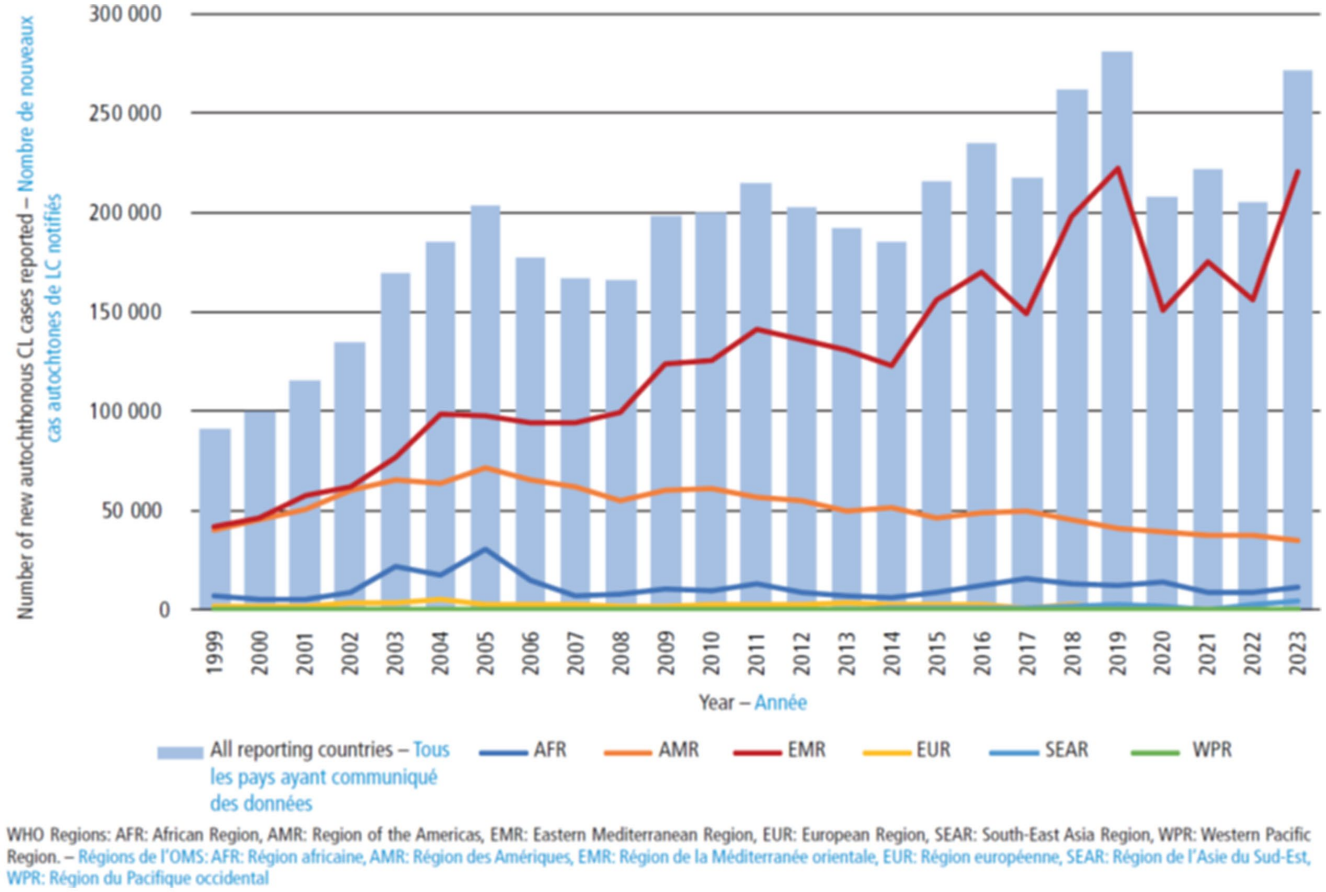
Evolution of the number of cutaneous leishmaniasis cases, by WHO region, 1999–2023. Reproduced from Jain et al. (2024).

Leishmaniasis is endemic in more than 90 countries, with the highest burden present in the Middle East, Northern Africa and Latin America. It is estimated that 600,000 – 1 M new cases occur each year (Figure 1). However, the geographic distribution is rapidly evolving due to climate change, deforestation and global warming, factors that facilitate sandfly habitat extension and contact with humans. Historically endemic in low-income regions in the tropics, CL has become a public health challenge also in high-income countries. The data forecast the continued expansion into regions further away from the Equator, both northwards and southwards.

The clinical manifestations of CL range from self-healing ulcers to disfiguring lesions when the parasite migrates from the site of infection into the mucosa of the nose, mouth, and throat (MCL). While rarely fatal, the disease imposes a severe burden on affected individuals. Stigma, depression, and economic losses affect most of the patients, particularly in low-resource settings. It is estimated that over 40 million individuals may live with the long-term consequences of past CL infections. Although CL ultimately self-cures, it has a substantial morbidity due to the long presence of a skin ulcer, usually in a part of the body that is visible, and the psychosociological impact of disfigurement, which produces depression and oftentimes discrimination (Bern et al., 2008). In some regions of Latin America, the disease is associated with the forest-dwelling guerrilla, adding a layer of social discrimination.

An inherent difficulty with using anti-VL drugs for CL is that the benefit may outweigh the toxicity of systemic agents for a fatal disease such as VL, but such toxicity may be harder to justify for a non-fatal disease such as CL (Murray et al., 2005). As a result, treatment of CL is unsatisfactory and is currently the major unmet medical need for the leishmaniases. Treatment of the disease depends on the parasite species, the host, the endemic region, socioeconomic status, and the availability of medical care.

Being CL not a life-threatening disease, its prevention and treatment is not given the priority it should have. CL remains an unpreventable disease, with no vaccine still available for humans, therefore novel therapies are urgently necessary. A new approach for CL treatment favored by the World Health Organization (WHO) and other experts is the use of a local treatment followed by parenteral treatment only if the local treatment fails or cannot be performed (González et al., 2008; Pinart et al., 2020). Treatment options are limited, with existing therapies being either toxic and painful or less toxic and easier to administer but prohibitively costly or inaccessible. Topical treatments for CL are particularly interesting due to their potential to be efficacious, and less toxic/painful than systemic treatments. They could improve patient compliance and allow self-treatment, diminishing associated financial costs both for the patients and for the states that usually provide the treatments.

Local treatments such as photodynamic, thermal and cryotherapy have proven to be efficacious alternatives to systemic treatments in some cases. These treatments have been reviewed elsewhere (Garza-Tovar et al., 2020; Orabi et al., 2023). In this work we focus on topical treatments for CL. We discuss epidemiology of the disease and availability of treatments and then center on topical treatments, covering advances in preclinical and clinical studies.

## 2 Epidemiology

Neglected tropical diseases (NTDs), including CL, have shown a rising global incidence since 2012 (Ruiz-Postigo et al., 2023; IHME, 2024; GBD, 2019). Since they are predominantly disablers rather than killers, their burden is best measured using DALYs (years of healthy life lost to premature death and disability, estimated from incidence and prevalence data). This metric however still overlooks the economic and psychosocial impacts of the disease. The real burden of the disease is difficult to estimate, with studies such as the Global Burden of Disease being criticized for underestimating reports (Bailey et al., 2017). Leishmaniasis ranked as the fourth most serious NTD in terms of DALYs in 2010 (Hotez et al., 2014), with tegumentary leishmaniasis (CL and MCL) experiencing the greatest DALYs increase among NTDs between 1999 and 2023 (Herricks et al., 2017).

CL’s epidemiological burden is significant, with 45% of reporting countries now considered endemic. The disease is concentrated in the Americas, Eastern Mediterranean, North Africa, and East Africa regions and is increasingly affecting children (WHO 2024; Knight, 2023; Haftom et al., 2020; PAHO, 2024b). Global case numbers have steadily risen since 1999, peaking in 2019 before declining during the COVID-19 pandemic. By 2023, cases had returned to pre-pandemic levels (Figure 1) (Jain et al., 2024). The highest incidence of NTDs has been historically observed in tropical and sub-tropical (mostly low-income) regions of the planet (Stolk et al., 2016; Collis et al., 2019). Between 1990 and 2019, higher income regions experienced increasing incidence, whereas low-middle income regions remained stable and low-income regions presented a decreased incidence (Ruiz-Postigo et al., 2023; European Centre for Disease Prevention and Control, 2015). In 2023–2024 the global trend was mainly due to the trend in the Eastern Mediterranean Region (Figure 1), although there is a significant underreporting in Europe, the Western Pacific, and China (Ruiz-Postigo et al., 2023; WHO, 2024). Tegumentary leishmaniasis has an equal impact in females and males (Ruiz-Postigo et al., 2023; Scheufele et al., 2020; Jain et al., 2024).

Infected animals, sandfly habitats and locally infected patients are now present in new regions. Climate change, global warming and displacement of people due to conflict are critical drivers of CL’s spread (Githeko et al., 2000; Du et al., 2016; López et al., 2016). In the last ten years, autochthonous cases have been reported in the USA (Texas, Oklahoma, Florida and Arizona), East Europe, and Southern Europe, where the disease is recognized as endemic (GHO, 2024; WHO, 2024; Scheufele et al., 2020; Stolk et al., 2016; Maia et al., 2023; Hotez, 2018; Clarke et al., 2013; McIlwee et al., 2018). Also in these high-income regions, the disease is prevalent among people living in poverty. This is not a negligible amount of the population, reaching more than 35% of children in the state of Texas, USA (Hotez, 2018). A northwards trend of expansion of the disease has been observed both in Europe and in North America (Wright et al., 2008; Kipp et al., 2020; Clarke et al., 2013; McIlwee et al., 2018; Scheufele et al., 2020; Maia et al., 2023). Domestic dogs are an important reservoir of leishmaniasis (Abuzaid et al., 2020). Now, roughly 10% of dogs in Southwestern Europe are carriers of canine leishmaniasis, and forecasting models predict continued northward expansion of sandfly distribution into Southern Canada and Northern Europe (Koch et al., 2017; González et al., 2010). The same trend is observed in the Southern Hemisphere, with leishmaniasis cases in dogs appearing for the first time in history in previously disease-free central Argentina (García Campos et al., 2024; Ministerio de Salud de la Provincia de Córdoba, 2023; Visintin et al., 2016).

The psychosocial and economic consequences of CL are profound, with over 40 million individuals potentially living with long-term effects of infection (Bailey et al., 2017). Despite these burdens, tools for large-scale control remain inadequate, and under-recognition of disease prevalence continues to skew estimates in global health studies (Mitra and Mawson, 2017).

## 3 Availability and cost of leishmaniasis treatments

Leishmaniasis is associated with poverty, even in high-income countries, and perpetuates the cycle of poverty and disease (Wijerathna et al., 2018). The financial impact on healthcare systems and patients is substantial. The global market for anti-leishmaniasis drugs, valued at $4 to $7 billion annually, faces challenges such as limited funding, reliance on sole suppliers, and supply shortages (Choi et al., 2021). Table 1 shows the estimated cost of leishmaniasis treatment in different regions. Costs include medication and direct treatment expenses. Drug costs constitute over 60% of the total treatment expenses across all therapies, and vary widely when comparing WHO negotiated prices, prices resulting of an agreement with a pharmaceutical company or actual market prices or the drugs. Although there is a wide variety of treatments, they remain largely inaccessible in endemic countries: their prices are high and highly variable; many products lack approval by the regulatory authorities, the existence of a single manufacturer hinders price negotiations (liposomal Amphotericin B and miltefosine). Donor dependency and lack of (reliable) local manufacturing further complicate the issue. Finally, the limited infrastructure of health systems in endemic countries hinders the large-scale implementation of treatments.

**Table 1:** Comparison of estimated cost of leishmaniasis treatment in different regions.

Region	Estimated cost per patient (USD)	Notes	References
Africa	$214 - $636	Most drug prices subsidized by WHO	Tessema et al. (2024), Tachfouti et al. (2016), and Sunyoto et al. (2019)
Americas	$142 - $12,000	Some drug prices subsidized by WHO or PAHO	de Assis et al. (2017), Cardona-Arias et al. (2018), Rodriguez et al. (2019), Cardona-Arias et al. (2017), Tejada Caminiti (2018), Carvalho et al. (2024), Brito et al. (2019), PAHO (2024a)
South-East Asia	$150 - $1,000	Some drug prices subsidized by WHO	Refai et al. (2017), Refai et al. (2017), Okwor and Uzonna (2016)
Eastern Mediterranean	$150 - $1,500	Some drug prices subsidized by WHO	Salimi et al. (2019) and Khajedaluee et al. (2024)

Efforts to control leishmaniasis have been supported by various international initiatives, notably the Leishmaniasis East Africa Platform (LEAP) and ASCEND (Accelerating the Sustainable Control and Elimination of Neglected Tropical Diseases). The LEAP was created in 2003 in Sudan by the Drugs for Neglected Diseases Initiative (DNDi) to support training, the generation of scientific evidence, and to distribute new treatments for leishmaniasis in East Africa (DNDi, 2024). ASCEND was a UK governmental program that operated in 25 countries across Africa and Asia, addressing NTDs. Its main objective was to strengthen national health systems to ensure the control and eventual elimination of these diseases (Rowan et al., 2022). Initially, the program was set to run until 2022, but in April 2021, the UK government reduced its funding (Davies, 2022). After ASCEND’s withdrawal, in August 2021, the END Fund (Ending Neglected Diseases), with support from ELMA Philanthropies, funded four organizations to continue initiatives in five East African countries. However, the disruption of ASCEND impacted service delivery in some healthcare facilities, while reliance on expensive donor-funded medications further worsened the situation (Davies, 2022). An international organization that plays a crucial role in ensuring the availability of essential medicines in endemic countries of the Americas is the Pan-American Health Organisation (PAHO). Its Strategic Fund aims to facilitate more equitable access to treatments. Through negotiations with various pharmaceutical companies, medications can be obtained at competitive prices (PAHO, 2025; 2024a; Vlassoff et al., 2023). PAHO also facilitates equipment for local treatments such as thermotherapy (PAHO, 2023; PAHO, 2025; Tejada Caminiti, 2018).

### 3.1 Topical sodium chlorite

The electrosurgical cauterisation to clean the wound followed by a moist wound treatment with 0.045% sodium chlorite solution (Leaflet LeiProtect of Weisenmedizin e.V., 2021) has shown good results in clinical trials against *L. tropica and L. major* (see section 6) but is unfortunately only available in Germany. The nonprofit organization Waisenmedizin e. V. (Freiburg, Germany) holds a license which has been approved by the German Federal Institute for Drugs and Medical Devices (Bonn, Germany) (Leaflet LeiProtect®). The approval was temporary (prolonged until September 30, 2021) and so far only valid for the treatment of CL within Germany (Debus et al., 2022). A lack of funds to obtain the necessary certificate from EU authorities seems to impede the export of the treatment to endemic countries.

### 3.2 Pentamidine

Pentamidine has antifungal and antiprotozoal activity (Piccica et al., 2021). One of its main advantages is its affordability, compared to other treatments such as miltefosine and liposomal amphotericin B (L-AmB). However, it has fallen out of favor in recent years due to the availability of other options and its associated side effects (Piccica et al., 2021; Carvalho et al., 2024). Most treatment guidelines however still recommend pentamidine for *L. guyanensis* and *L. panamensis*, causative agents of CL and MCL (PAHO, 2022).

Pentamidine isethionate has not been approved for protozoal infections such as leishmaniasis in the USA. Nevertheless, the Centers for Disease Control and Prevention recommends its use for leishmaniasis treatment (Hafiz and Kyriakopoulos, 2023).

### 3.3 Paromomycin and Imiquimod

Paromomycin (PM) is an antibiotic shown to have leishmanicidal activity (El-On et al., 1986) against *L. panamensis, L. braziliensis and L. major* (Sosa et al., 2019; Soto et al., 2019; Ben Salah et al., 2013) and can be used in i.v., i.m. and topical forms. The most frequent side effects of systemic PM are ototoxicity, local pain, erythema, vesicles and skin irritation (Matos et al., 2020). Patents covering the compound, topical creams, and later oral formulations have expired.

Different types of topical formulations of PM have been studied, including the combination with gentamicin, methylbenzethonium, and urea (Matos et al., 2020; Armijos et al., 2004). In topical preparations, the combination of PM with methylbenzethonium is available only in Israel as an ointment (Leshcutan®, Teva Pharmaceuticals). A second topical formulation combining paromomycin with urea is also only available outside the International Conference of Harmonization zone (ICH for USA, EC, and Japan).

The cream containing PM and gentamicin has proven to be very efficacious (see section 6 on Clinical Trials). This formulation (Combo Cream) was initially produced by Teva Pharmaceuticals and the US Army but was later (2019) licensed by APPILI Therapeutics (Canada). After this, and due to the change in the manufacturing company, the FDA requested new data for approval of the product. In 2024 APPILI announced that the FDA had agreed to use an *in vitro* assay (instead of the immensely expensive new preclinical and clinical data) to compare their product with that tested in the clinical trials. Sadly, to this day this treatment remains unavailable, apparently depending on the company obtaining more funding (Appili Therapeutics, 2024).

Imiquimod 5% cream is widely available under various brand names, and as a generic medication since 2015. It is indicated for the topical treatment of genital warts and some forms of skin cancer, but has also been proven to have some efficacy against CL, see Section 6.

### 3.4 Azoles

Azoles such as ketoconazole, fluconazole, and itraconazole have been used for the treatment of leishmaniasis (Sousa et al., 2011; Saenz et al., 1990; Navin et al., 1992). The second edition of the Guidelines for the treatment of leishmaniasis published by the PAHO recommends the use of imidazoles, although ketoconazole has been removed from the list of therapeutic options (PAHO, 2022; Aronson et al., 2016). Regarding safety, the FDA warns that use of ketoconazole carries a risk of liver damage and adrenal gland problems (FDA, 2016).

### 3.5 Pentavalent antimonials

Pentavalent antimonials are the first-line treatments recommended by the WHO for leishmaniasis and are widely available. These compounds were developed and introduced in the early 20th century, and all related patents have long expired. These drugs are sodium stibogluconate (Pentostam®), administered i.v. or i.m., and meglumine antimoniate (MA, Glucantime®), administered i.v. or intralesionally (Heras-Mosteiro et al., 2017; PAHO, 2022). They are also produced as generic products by a limited number of manufacturers, primarily in endemic regions (Brazil, India, Sudan) and through public sector or nonprofit channels.

Systemic pentavalent antimonials are associated with a range of adverse effects, including local pain, nausea, vomiting, weakness, myalgia, abdominal colic, hepatotoxicity, renal toxicity and, most critically, cardiotoxicity (Frézard et al., 2009). They are contraindicated in individuals with drug sensitivity, a history of cardiovascular disease, certain chronic conditions, young children, and pregnant or breastfeeding women (Ministerio de Salud Presidencia de la Nación 2010).

Intralesional pentavalent antimonials have been recommended by the PAHO and the WHO as a treatment for uncomplicated localized CL (PAHO, 2022). This treatment offers advantages such as a shorter duration (5 days) and fewer side effects, although it can produce pain and vasovagal shock due to severe pain (De Oliveira Duque et al., 2016). This treatment also requires specifically trained personnel, absent in many regions. The increasing failure rate, probably linked to drug resistance, is other very important disadvantage.

Although these treatments are considered traditional, they have not been approved by the FDA and are not commercially available in the United States. MA is only accessible through an individual Investigational New Drug protocol authorized by the FDA (Centers for Disease Control and Prevention, 2024).

### 3.6 Amphotericin B

The second-line treatment for leishmaniasis involves the i.v. administration of amphotericin B (AmB). Four formulations are commercially available: amphotericin B deoxycholate (one of the most commonly used due to its availability and low price), liposomal amphotericin B (L-AmB), AmB cholesterol dispersion, and AmB lipid complex. The active pharmaceutical ingredient, amphotericin B, was discovered in the 1950s and is long off-patent globally.

Although AmB deoxycholate is effective, its side effects are more severe than those of the other formulations (Lindoso et al., 2012). This systemic treatment has not been approved by the FDA for leishmaniasis (Centers for Disease Control and Prevention, 2024). Nevertheless, the PAHO recommends it for cutaneous leishmaniasis (PAHO, 2022). The liposomal formulation was approved by the FDA in the United States only for the treatment of VL (Meyerhoff, 1999).

L-AmB has replaced the deoxycholate formulation in most high-income countries due to its lower adverse effects. The most widely known branded version is AmBisome®, which was originally developed by Nexstar Pharmaceuticals, later acquired by Gilead Sciences. The original formulation patent has expired, but the process and delivery patents have lasted until 2016. It remains uncommon in low- and middle-income countries due to its cost. AmBisome® prices vary widely worldwide (Lee et al., 2024). After negotiations, a reduced price of USD 16 per vial was offered in certain low- and middle-income countries, but prices continue to rise year after year. The generic product is produced by companies in India and China but expanding competition in the L-AmB market is crucial for equitable access (Lee et al., 2024). Clinical applications of L-AmB for CL have shown variable efficacy against different *Leishmania* strains and clinical manifestations, and its activity against various species is still under evaluation (Ferreira et al., 2024; Senchyna et al., 2020).

### 3.7 Miltefosine

Miltefosine is the only oral treatment available for the disease. Initially developed to be used as an oral treatment against cancer, it was approved in India to be used in VL cases in 2002. In 2014 the FDA approved it for the treatment of cutaneous, mucosal, and VL (Centers for Disease Control and Prevention, 2024). Miltefosine is used for all forms of leishmaniasis, but it has reproductive toxicity effects both in females and males, requiring contraception in women of childbearing age (PAHO, 2022; Jung et al., 2024). This makes the use of the treatment extremely inconvenient in low socioeconomical status female patients. Other commonly observed side effects are gastrointestinal, including nausea or vomiting, abdominal pain, and diarrhea (Astman, 2024; LiverTox: Clinical and Research Information on Drug - Induced Liver Injury (Internet), 2017).

Despite being on the WHO’s Essential Medicines List since 2011, its high cost and limited access hinder widespread use. Miltefosine’s compound patent has expired globally, and it is available in generic form. The patent expired roughly around 2002–2004, and there exist generic manufacturers in India and Bangladesh. Quality however varies among different producers, and supply chain inefficiencies, regulatory barriers, and counterfeit products further exacerbate access issues. Miltefosine is not registered in any country in South America. It can be obtained through the WHO, but only in a case-by-case bureaucratic process that forces extra work on the attending physicians (Sunyoto et al., 2018; Choi et al., 2021; Carvalho et al., 2024). Despite ongoing efforts by PAHO and WHO to negotiate price reductions and expand access, miltefosine continues to command a premium cost, limiting its affordability in many endemic settings.

### 3.8 Non-pharmacological local treatments

Non-pharmacological therapies, such as cryotherapy, photodynamic therapy, and thermotherapy, are useful, particularly for pediatric and pregnant patients. These treatments are advised for patients with no more than 1–3 lesions, each up to 900 mm^2^, avoiding areas like the face, skin folds, or joints (PAHO, 2022; Pradhan et al., 2022). While the therapies themselves are cost-effective, the required equipment can be expensive, and trained personnel are necessary to maintain the recommended protocols to ensure efficacy and safety.

Cryotherapy typically involves the application of liquid nitrogen, which destroys affected tissues. The effectiveness varies depending on the parasite species and dosing regimens (Salmanpour et al., 2006; Negera et al., 2012; Ranawaka et al., 2011; Layegh et al., 2009; Asilian et al., 2004; Mosleh et al., 2008). Common side effects include vesicle formation, erythema, swelling, burning pain, and both hypo- and hyperpigmentation in the treated skin (Mosleh et al., 2008; Ranawaka et al., 2011).

Thermotherapy encompasses various techniques, including hot water baths, laser therapy, ultrasound, infrared light, microwaves, radiofrequency and photodynamic therapy (Man et al., 2022; Safi et al., 2012; Cabral et al., 2024). The PAHO recommends thermotherapy for some patients with localized CL (PAHO, 2022). The FDA has approved a device named ThermoMed for thermotherapy procedures (Aronson et al., 2016). While the FDA has approved photosensitizing agents for the treatment of certain types of cancer, they are not approved for cutaneous leishmaniasis (The American Cancer Society Medical and Editorial Content Team, 2021).

## 4 Advantages of topical treatments

Leishmaniasis has been considered a set of distinct syndromes due to the wide range of parasite species involved, each of which presenting unique clinical manifestations, which affects the success of treatments (Mann et al., 2021). As already mentioned, the first line of treatment with pentavalent antimonials stand out for its toxicity, its painfulness and its contraindications to women during pregnancy and lactation, to children, to people with hypersensitivity to the drug and in some chronic diseases (Frézard et al., 2009; WHO, 2024). Intralesional (IL) administration is also associated with several side effects (De Oliveira Duque et al., 2016) and is very painful, leading the patient to abandon treatment (low compliance). Local therapies such as cryotherapy, thermotherapy and phototherapy are recommended alternatives but with limited use due to their variable effectiveness, the cost of the equipment and their unsuitability for lesions located on the face, skin folds or joints (Cardona-Arias et al., 2018).

Topical formulations offer several advantages over traditional therapies. One of the main benefits is the reduction of systemic toxicity, as they are applied directly and only to the skin lesions, limiting the drug’s absorption into the body and its distribution. Additionally, these formulations eliminate the need for painful injections, reducing the likelihood of patient dropouts. Topical therapies are also easier to administer, as they can be self-applied without the need for frequent visits to healthcare centers. This is particularly advantageous in resource-limited regions where these services are distant, reducing costs for patients and their families. Moreover, the production cost of a topical treatment is lower than that of a parenteral treatment, which should result in a more affordable market price for the formulation. These features make topical formulations an attractive option for managing the disease.

## 5 Preclinical studies of topical formulations

Several animal species such as mice, rats, hamsters, dogs, and non-human primates play an important role as animal models in the study of novel therapies for CL, with each model having its advantages and disadvantages (see Table 2). Although there is no validated animal model for CL, rodents (particularly BALB/c mice) are the preferred species. Their use has important advantages. The availability of strains with standardized or modified genetics, which eliminates the noise of a genetically heterogeneous background, is extremely useful. The lower cost of developing, breeding and conducting studies facilitates mice use in almost any laboratory, which is particularly important when the budget represents a limitation, usually the case in countries where the disease is endemic. Besides, although some of the observations made in rodents might not be similar to human hosts (inbred strains vs. genetic diversity in humans, molecular differences altering drug effects, and different immune responses), they provide a fast turnaround during the drug research process.

**Table 2:** Animal models used in leishmaniasis research.

Animal model	Advantages	Disadvantages	Leishmania species studied	References
Mice (*Mus musculus*) and Rats (*Rattus norvegicus*)	Genetic similarity to humans, rapid breeding cycles, minimal living space, cost effectiveness. Availability of numerous genetically homogeneous inbred strains, possibility of producing genetically engineered strains.	Lack of the genetic diversity found in human populations. Different genetic backgrounds show different immune responses. Different drug metabolisms and pharmacokinetics from humans.	*L. major, L. donovani, L. infantum, L. mexicana, L. tropica, L. amazonensis, L. braziliensis*	Terabe et al. (2004), Hartung (2024), Papadopoulou et al. (2002), Carrara and Stolf (2024), Velasquez et al. (2016), Tripathi et al. (2007), Murray (2001), Pérez-Cabezas et al. (2019), Buxbaum and Scott (2005), and Howard et al. (1980)
Hamsters (*Mesocricetus auratus*)	Used for strains of *Leishmania* with a low infectivity to mice. Considered the adequate bio-model for immunopathogenesis, drug discovery and vaccine development studies.	Scarcity of specific reagents and techniques to allow the study of the immunopathogenesis of the disease, which is crucial for the generation of therapeutic and vaccine targets	*L. braziliensis, L. amazonensis,* *L. guyanensis, L. panamensis, L. donovani, L. lainsoni,* *L. peruviana*	Robledo et al. (2012), Loria-Cervera and Andrade-Narváez (2014), Gomes-Silva et al. (2013), de Paula Freire et al. (2022), Ribeiro-Romão et al. (2014), and Banerjee et al. (2011)
Dogs (*Canis familiaris*)	Main reservoir of *Leishmania* in many countries. Immune response is highly similar to humans. Environmental homology with humans. Useful in the development of new diagnostic methods and control measures against the infection.	Cutaneous leishmaniasis has been little studied in domestic dogs and is poorly described. The same inoculation procedure induces different clinical patterns of the disease	*L. braziliensis, L. guayanensis,* *L. panamensis, L. infantum,* *L. mexicana, L. donovani, L. tarentolae*	Loria-Cervera and Andrade-Narváez (2014), WHO (2010), Cruz-Chan et al. (2014), Moreno and Alvar (2002), Cruz-Chan et al. (2014), Moreno and Alvar (2002), Mendoza-Roldan et al. (2024), and Konno et al. (2022)
Non-human primates, eg. *Chlorocebus, Macaca mulatta, Aotus Trivirgatus, Saimiri sciureus, Callithrix jacchus jacchus, Presbytis entellus, Cercopithecus aethiops*	Similarities to humans in anatomy, immunology and physiology. Used for assessing the pathogenicity related to infectious diseases, immune response, development of vaccines and drug therapies	Expensive and difficult to obtain and to handle. It is used as the final experimental animal in studies of safety and efficacy of vaccines and drugs	*L. major, L. infantum,* *L. braziliensis, L. amazonensis, L. mexicana, L. guyanensis, L. lainsoni, L. panamensis, L. donovani, L. chagasi*	Loria-Cervera and Andrade-Narváez (2014), André et al. (2020), Samant et al. (2021), Porrozzi et al. (2006), Freidag et al. (2003), Chapman et al. (1981), Broderson et al. (1986), Dennis et al. (1986), Binhazim et al. (1993), Gicheru et al. (1995), and Porrozzi et al. (2006)

Classic laboratory strains like BALB/c, C57BL/6 J, DBA/2, C3H/HeN, STS, and recombinant congenic mice have been extensively employed in CL research. The results consistently highlight the complex interplay between host/parasite interaction and genetics, vector factors, and environmental conditions. To increase the translational value of data obtained from mouse models, an extra step—adding a layer of complexity to experimental design—could prove beneficial. This might involve using murine models with diverse genetic backgrounds (e.g., wild-derived strains), including factors associated with the vector (e.g., mosquito saliva components), or co-housing with non-specific pathogen free mice (such as pet store mice) to better mimic the complexity of human infection (de Oliveira et al., 1999; Lipoldová and Demant, 2006; Kobets et al., 2012; Soong et al., 2012; Loria-Cervera and Andrade-Narvaez, 2014; Loureiro Salgado et al., 2022). Also, the use of the novel bioengineered human tissue/organoids which can show a high clinical mimicry (Loewa et al., 2023) shows important promise.

In Table 3 we compile some of the research carried out in the mouse model during the last five years to tackle CL with a topical approach. The aim of this section is to highlight what we identify as trends in CL therapies but is not an exhaustive revision, which can be found elsewhere (Lafleur et al., 2024; Maza Vega et al., 2023; Afonso et al., 2023). We identified two streams of research, the first looking to improve the efficacy of traditional drugs through carriers, enhancers or combined-drug therapy, while the second identifies compounds with antileishmanial activity, whether they are readily available in the market (repurposed drugs) or waiting to be discovered in nature (natural compounds). To improve the efficacy of traditional drugs the strategy is based on the implementation of combined therapies looking for synergistic effects of drugs, or the use of immunomodulatory agents to enhance the efficacy/toxicity ratio. The search takes advantage of novel nano-drug delivery systems that can transport antileishmanial drugs to their target organ, reducing adverse effects. Regardless of the approach chosen, the major issue identified from these studies is the lack of standardization (i.e., the size of inoculum, the site and method of inoculation and the starting treatment time) in experimental designs, which makes the comparison between the findings of different groups difficult.

**Table 3:** Preclinical studies of topical treatment for CL in mice.

Reference	Species	Inoculum size	SOI	Therapy	SOT	Schema	FU (after the EOT)	Outcome
Traditional drugs								
Jaafari et al. (2019)	*L. major*	2e6	Tail base	Liposomal-AmB	28 dpi	Twice a day for 28 days	28 days	Parasites were cleared from the lesion and spleen.
Nguyen et al. (2019)	*L. mexicana* and *L. major*	1e7	Tail base	AmB-DMSO after Microneedling	35 dpi (*L. mexicana*), and 21 dpi (*L. major*)	Once daily for 10 days	20 days for *L. mexicana* and 14 days for *L. major*	The treatment was ineffective.
Mostafavi et al. (2019)	*L.major*	2e6	Footpad	AmB-Glucantime Niosomal gel	28 dpi	Twice daily for 30 days	26 days	The therapy was superior to Glucantime.
Dar et al. (2019a)	*L. mexicana*	5e6	Tail base	AmB-Milt co-loaded SGUDLs	28 dpi	Twice daily for 28 days	0 days	Complete resolution of the lesion and lower PL than AmB gel treatment.
Alves et al. (2020)	*L. major*	1e6	Tail base	AmB-GA and AmB-EA	40 dpi	Twice daily for 21 days	14 days	Therapies were superior at reducing PL than AmB.
Fernández-García et al. (2020)	*L. amazonensis*	2e7	Tail base	AmB-TFs	35 dpi	Once daily for 10 days	11 days	Therapy was as effective as Glucantime (IL) at reducing PL.
Riaz et al. (2020)	*L.major*	4e7	Tail base	AmB-NLCs	14 dpi	10 days	1 day	Therapy showed the same efficacy as liposomal AmB (IV).
López-Arencibia et al. (2024)	*L. amazonensis*	1e6	Tail base	AmB-Sepigel	35 dpi	Once daily for 14 days	28 days	Therapy was less effective than oral Milt and IP MA.
López-Arencibia et al. (2024)	*L. amazonensis*	1e6	Tail base	MA-Sepigel	35 dpi	Once daily for 14 days	28 days	Therapy was less effective than oral Milt and IP MA.
Neira et al. (2019)	*L. braziliensis and* *L. panamensis*	2e6	Tail base	Milt gel	60 dpi	Once daily for 20 days	15 days	The treatment reduced LS by 84–100%, with no detected parasites on smears or biopsies.
Dar et al. (2019b)	*L. mexicana*	5e6	Tail base	Milt-APG co-loaded SGNTs gel	21 dpi	Twice daily for 28 days	0 days	Therapy resulted in complete resolution of the lesion.
Kavian et al. (2019)	*L. major*	4e6	Tail base	Liposomal Milt	28 dpi	Twice daily for 28 days	28 days	Reduction in LS with no relapse. No effect in PL.
Peralta et al. (2021a)	*L. amazonensis*	2e7	Tail base	Milt and Milt-FL	35 dpi	Twice daily for 21 days	28 days	Therapy showed complete cure with no relapses.
Batool et al. (2021)	*L. major*	NR	Ear pinnae	Milt loaded TF gel	14 dpi	Once daily for 42 days	0 days	Therapy showed 7-fold reduction in PL and LS compared to untreated control.
Natural compounds								
Malli et al. (2019)	*L. major*	1e6	Tail base	Cs-NPs	63–77 dpi	Once daily for 21 days	14 days	Cs-Coated PIBCA NPs showed better results than AmB-DOCs (IL).
Sosa et al. (2020)	*L. amazonensis*	5e3	Ear pinnae	(−)-EGCG	42 dpi	Once daily for 18 days	7 days	Therapy showed a similar efficacy to Glucantime (IP).
Sharma et al. (2023)	*L. major*	1e7	Footpad	Farnesol ointment	30 dpi	Twice daily for 10 days	10 days	Therapy showed an efficacy comparable to topical PM. Complete healing.
Sahebi et al. (2024)	*L. major*	2e6	Tail base	CUR-NE	28 dpi	Once daily for 21 days	28 days	The therapy showed an efficacy comparable to the AmB (IL).
Martínez-Salazar et al. (2020)	*L. major, L. major Seidman, L. mexicana*	1e5	Ear dermis	MF29	12 dpi	Once or twice daily for 28 days	9 days	Limited efficacy, incomplete healing
Alemzadeh et al. (2022)	*L. major*	2.5e6	Tail base	AgNPs *A. aucheri, quercetin*	21–28 dpi	Once daily for 21 days	0 days	Limited efficacy, incomplete healing
Clemente et al., 2024	*L. braziliensis*	1e8	Dorsum dermis	QUE, CUR, and PIP	n.d.	Once daily for 14 days	76 days	Complete healing with QUE and CUR, limited efficacy of PIP
Kawakami et al. (2021)	*L. amazonensis*	1e6	Tail base	SO-NE	42 dpi	Twice a day for or 30 days	0 days	Very limited efficacy
Repurposed Drugs								
Niroumand et al. (2024)	*L. major*	NR	Tail base	ART-Niosomal gel (Antimalarial)	After ulcerated lesion development	Once daily for 28 days	28 days	Therapy was superior to nanoliposomal AmB gel.
Peralta et al. (2021b)	*L. amazonensis*	1.5e7	Tail base	Ris and EuE-Ris (Bone resorption inhibitor)	35 dpi	Twice daily for 22 days	0 days	The EuE-Ris led to lesion cicatrization and a slightly higher reduction in the PL than Ris alone.
El-Dirany et al. (2022)	*L. major*	1e5	Tail base	19–2.5 and 19-4LF (Synthetic Antimicrobial Peptide)	56 dpi	Twice daily for 30 days	3 days	Therapies showed a significant reduction in skin PL compared to negative control.
De Lima et al. (2023)	*L. amazonensis*	1e6	Tail base	8-HQ (Fungicide, Antibacterial)	42 dpi	Once daily for 14 days	7 days	Therapy showed an efficacy comparable to Glucantime (IL).
Nahanji et al. (2024)	*L. major*	NR	Tail base	FZL-NE (Antifungal)	After ulcerated lesion development	Every other day for 28 days	28 days	Therapy showed an efficacy comparable to Glucantime (IP).

SOI, site of injection; SOT, start of treatment; FU, Follow-up; EOT, end of treatment; IL, intralesional; IV, intravenous; PL, parasite load; LS, lesion size; dpi, days post-infection; OA, oleic acid; GA, gallic acid; EA, ellagic acid; SGUDL, second generation ultra-deformable liposome; SGNT, second generation nano-transferosome; APG, apigenin; TF, transferosome; NLC, nanostructured lipid carrier; NE, nanoemulsion; PG, propylene glycol; OSAL, octyl salicylate; DMI, dimethyl isosorbide; FL, flexible liposome; Milt, Miltefosine, AmB, Amphotericin B; AmB-DOC, amphotericin B-deoxycholate; PM, paramomycin; MA, meglumine antimoniate; Cs-NP, chitosan coated nanoparticle; PIBCA, poly(isobutylcyanoacrylate); EGCG, epigallocatechin gallate; CUR-NE, curcumine nanoemulsion; ART, Artemether; Ris, risedronate; Eue-Ris, eudragit EPO-risedronate; AAP, Antibacterial Agent Peptide; 8-HQ, 8-hydroxyquinoline; FZL, fluconazole; NR, non reported; MF29, (ethyl 3-(2-chloroacetamido) benzoate); QUE, quercetin; PIP, piperine; SO-NE; Sucupira oleoresin nanoemulsion.

Topical miltefosine formulations and some repurposed antifungals show very good promise. Combination therapies have proven very useful in some cases and should also bring good results (Van Griensven et al., 2024). Difficulties related to the production hinder the emergence of new effective natural compounds; despite this, they are a promising way forward, as shown by the very good efficacy of compounds such as quercetin and farnesol (Afonso et al., 2023). Also, the search of new natural compounds by hight-throughput techniques will surely broaden the spectrum of efficacious molecules (Dantas et al., 2022; Khan et al., 2024).

## 6 Clinical studies of topical treatments

There is an important academic production of preclinical studies on the subject of topical treatments. A most important obstacle to the translation of these results are the high costs involved and the lack of interest of big pharmaceutical companies. For example, a search of ClinicalTrials.gov (accessed February 24^th^, 2025) with the keywords “cutaneous leishmaniasis” + “topical administration” retrieves 27 entries. All of them are sponsored by universities, state-owned research facilities or NGOs (Non-Governmental Organizations). In general, and most likely due to a lack of stronger funding, the studies do not comprise multi-centered studies with large numbers of patients. We summarize below clinical results on those topical treatments that have been more thoroughly studied. For a systematic comparison, see Table 4.

**Table 4:** Clinical trials on topical treatments.

Treatment(s)	Type of clinical trial (blinding)	Nr. of patients	Placebo control	Follow up ^#^	% of treated patients cured (% cured with placebo/ comparison treatment)	Statistical difference cure treatment vs. placebo/comparison treatment	Observations	Region	References
Paromomycin									
paromomycin 15% + gentamicin 0.5% vs. vehicle	Phase 2, single center, pilot (investigator)	45	Yes	180 days	61% (55%)	No	lesion cure time significantly faster in treatment arm	New World	Soto et al. (2002)
paromomycin 15% + gentamicin 0.5% vs. vehicle	Phase 2, two centers (double)	92	Yes	180 days	94% (71%)	Yes	lesion cure time faster in placebo arm	Old World	Salah et al. (2009)
paromomycin 15% + gentamicin 0.5% vs. paromomycin 15%	Phase 2, one center (double)	30	No	168 days	87% (60%)	Yes	cure time equal in both treatments	New World	Sosa et al. (2013)
paromomycin 15% + gentamicin 0.5% non-occlusive vs. occlusive	Phase 2, n.d. (investigator)	48	No	90 days	92% (79%)	No	Parasite loads reduced 55-fold (occlusive) and 77-fold (non-occlusive)	Old World	Salah et al. (2014)
paromomycin 15% + gentamicin 0.5% vs. paromomycin 15%	Phase 3, multicenter (double)	399	No	168 days	79% (78%)	No	lesion cure time faster in combined treatment	New World	Sosa et al., 2019
Amphotericin B									
Liposomal amphotericin B vs. IL meglumine antimoniate	Phase 2, single center (open)	110	No	180 days	56% (68%)	No		Old World	Layegh et al. (2011)
amphotericin B 3% 3 vs. 2 times per day	Phase 2, multicenter (open)	80	No	180 days	39% (35%)	No	efficacy does not support continuing with clinical development	New World	López et al. (2018)
Liposomal amphotericin B 0.4% for 7 or 14 days	Phase 1, single center (double)	27	Yes	7–14 days	n.d.	No	Safer if applied twice a day for 7 days	Old World	Eskandari et al. (2019)
Liposomal amphotericin B 0.4% vs. vehicle	Phase 2, pilot, single center (double)	13	Yes	56 days	Primary endpoint not achieved	No	PCR negative in 75% of treated vs. 89% of placebo patients	Old World	Horev et al. (2022)
Topical liposomal AmB 0.4% alone vs. i.m. MA + topical liposomal AmB 0.4% vs. IL MA + cryotherapy	Phase 2, pilot, single center (open)	66	No	42 days	95% (92–48%)	n.d.		Old World	Khamesipour et al. (2022)
Imiquimod									
i.m. MA + topical imiquimod	Phase 2, two centers (open)	12	No	180 days	90%	--	Patients had been unresponsive to i.m. MA	New World	Arevalo et al. (2001)
imiquimod 5% + MA vs. MA + vehicle	Phase 2, single center (double)	40	Yes	360 days	72% (75%)	No	Only included patients who had been unresponsive to MA. Imiquimod shortened time for cure	New World	Miranda-Verástegui et al. (2005)
Imiquimod 5% + MA vs. vehicle + MA	Phase 2, (investigator)	119	Yes	140 days	44% (48%)	No		Old World	Firooz et al. (2006)
imiquimod 5% + cryotherapy vs. IL MA	Phase 2, single center (open)	50	No	90 days	65% (83%)	No		Old World	Shamsi Meymandi et al. (2011)
IL SSG + imiquimod 5% vs. IL SSG + vehicle	Phase 2, single center, (investigator)	131	Yes	42 days	94% (74%)	Yes	Better scar quality with imiquimod	Old World	Dhaher and Hussein (2022)
GM-CSF									
Topical GM-CSF 0.01% + oral miltefosine vs. placebo + miltefosine vs. MA	Phase 2, two centers, (double)	133	Yes	180 days	76% (77–44%)	No	Healing time longer in the MA group	New World	Machado et al. (2020)
Topical GM-CSF 0.01% + oral miltefosine vs. placebo + miltefosine vs. MA	Phase 2, single center, (double)	150	Yes	180 days	58% (66–52%)	No	Part of previous study. No difference in healing times.	New World	Mendes et al. (2021)
Sodium Chlorite									
EC + Topical Sodium Chlorite 0.045% vs. EC + vehicle	Phase 2a, single center, (double)	135	Yes	180 days	90% cure in 74 (76) days	No	Primary outcome was time to heal	Old World	Jebran et al. (2014)
EC + sodium chlorite 0.045% vs. sodium chlorite 0.045% alone vs. IL SSG	Phase 2b, single center, (open)	87	No	180 days	100% (87–65%)	Yes		Old World	Stahl et al. (2014)

^#^After initiation of treatment. MA = meglumine antimoniate; IL = intralesional; i.m. = intramuscular; n.d. = no data; SSG = sodium stibogluconate; GM-CSF = granulocyte-macrophage colony-stimulating factor; EC = electrocauterization.

### 6.1 Topical Paromomycin

In topical preparations, paromomycin is a component of two antileishmanial products, containing methylbenzenthonium chloride and urea. The former had good efficacy but produced a high degree of local irritation (El-On et al., 1984; El-On et al., 1986; El-On et al., 1992, Soto et al., 1995; Arana et al., 2001); the latter was not efficacious enough compared to placebo (Neva et al., 1997; Iraji and Sadeghinia, 2005; Faghihi and Tavakoli-kia, 2003; Asilian et al., 1995).

A hydrophilic formulation (a cream containing more than 10 excipients) of 15% PM and 0.5% gentamicin (Combo Cream) has been studied in several clinical trials. It was developed at the Walter Reed Army Institute of Research, an institution based in the USA and financed by the US Department of Defense. Intended for the treatment of uncomplicated CL, it has been studied in randomized phase 1, 2, and 3 clinical trials. The trials were conducted both in New World (Colombia and Panama) (Soto et al., 2002; Sosa et al., 2019) and in Old World (Tunisia and France) (Ben Salah et al., 2009; Ben Salah et al., 2013) patients. In travelers (Mouri et al., 2023) and soldiers (Soto et al., 2002); in men (Soto et al., 2002), women and children (Ben Salah et al., 2009; Ben Salah et al., 2013; Sosa et al., 2019; Sosa et al., 2013). Children responded very well to the treatment (82–84% cure), which is especially important since this is a group with low adherence and lower cure rates when treated with parenteral medications.

Only two of the studies were performed against placebo. In the best of these studies (larger number of patients, double blinding, two centers) a higher percentage of patients were cured, and the cure was faster than in the placebo group (Soto et al., 2002; Ben Salah et al., 2009). In later studies, upon once or twice daily application for 20 days, the clinical cure of the index lesion for PM-gentamicin was 79% and for PM alone was 78%. Compliance was excellent (98%) and the efficacy was the same as that of pentavalent antimonials (Sosa et al., 2019; Salah et al., 2014). Other trials in the Americas showed in some cases showing superiority of the combination compared to PM alone, in other showing no superiority (Sosa et al., 2013; Sosa et al., 2019). Non-occlusive dressing seems to be better than using occlusion, and once daily treatment had the same efficacy as the twice daily scheme (Salah et al., 2014). A study of pharmacokinetics with CL patients from two phase 2 clinical trials, both in Latin America, compared 15% PM and 15% PM + 0.5% gentamicin. The percentage of dose absorbed on day 20 was approx. 12 and 10% for PM alone and combination, respectively. PM concentrations in plasma after 20 days of application were 5–9% of those after i.m. administration of 15 mg/kg of body weight/day to adults, indicating that effective topical treatment is possible with low systemic absorption, thus avoiding drug accumulation and toxicity (Ravis et al., 2013).

With adverse effects that are mild to moderate local irritation, the lesions become bigger at the beginning of treatment and cure after the 20-day treatment; the epithelialization is observed at days 35–42. No relapse is observed up to 180 days and the results are at least as good as with toxic traditional treatments. In summary, although most of the trials were single-centered and with a relatively small number of subjects, the evidence shows that both PM and the combination paromomycin+gentamicin cures 80–94% of patients with both Old or New World uncomplicated CL and can reduce parasite load in the deep dermis up to 77 times (Soto et al., 2002; Sosa et al., 2019; Ben Salah et al., 2009; Ben Salah et al., 2013). Gentamicin does not make the treatment more efficacious, but its presence makes the lesions cure faster (Sosa et al., 2019). Even though this treatment is arguably the best topical treatment developed for CL, it is, as discussed in Section 2, still unavailable.

### 6.2 Topical AmphotericinB

An AmB 3% w/v cream was studied in a trial sponsored by DNDi recently. In a phase Ib/II design, it compared the application of the cream either 3 or 2 times per day for 30 days. The treatment produced only mild local adverse events, but the efficacy was too low to continue clinical trials (López et al., 2018). A trial studying a gel formulation of L-AmB (0.4% w/v) efficacy against *L. major* produced only mild local irritation but the presence of parasites as measured by PCR at 56 days was the same for placebo and formulation (Horev et al., 2022).

A topical nano-liposomal formulation of 0.4% w/v AmpB (SinaAmpholeish®) has shown promising results. It produced only mild local reactions, with no difference between treatment and vehicle (Eskandari et al., 2019). This same formulation was later studied in Iran to evaluate safety and efficacy in patients with lesions produced by *L. major,* comparing: the standard national treatment (IL MA + cryotherapy) vs. daily i.m. MA plus topical L-AmB 0.4%vs. topical L-AmB 0.4% alone. Two of 36 patients reported a tolerable burning sensation with the topical treatment. Both the combination of national standard treatment with topical treatment and topical treatment alone showed much higher efficacy (92–95% cure) than patients who received only the national standard treatment (48%) (Khamesipour et al., 2022). A retrospective study of this same formulation (SinaAmpholeish®) was conducted in Iran, in regions where *L tropica* and *L major* are causative agents of CL. The treatment with only SinaAmpholeish® was compared to the combination of: SinaAmpholeish® plus Glucantime®; SinaAmpholeish® plus cryotherapy and SinaAmpholeish® plus Glucantime® and / or cryotherapy. 90–96% of patients had received Glucantime®, and so the number of patients treated only with the topical treatment was very low. Recurrence after treatment was found to be between 0.4 and 3%, with most patients having been cured (Alizadeh et al., 2023).

Despite being very efficacious when used in the parenteral form, AmB has proven difficult to formulate as an efficacious topical treatment. Except for the study by Khamesipour et al., 2022 showing 95% efficacy, the results are mixed. It may be possible however that an AmB topical formulation can be as effective as a much more painful IL treatment (Layegh et al., 2011), and more research is needed to find an optimal topical formulation.

### 6.3 Topical imiquimod

In 2001, a small group of antimoniate-resistant CL patients in Peru was treated by a combination of topical imiquimod 5% cream and i.m. administration of MA. A cure rate of 90% was achieved, indicating the possible overcoming of resistance by the addition of the topical treatment. A first attempt to use topical imiquimod alone had been of limited success (25% cure) (Arevalo et al., 2001).

Topical imiquimod 5% cream (Aldara®, 3 M Pharmaceuticals) was studied in a double-blind, randomized trial in subjects in Peru, for whom an initial course of antimony therapy had failed (Miranda-Verastegui et al., 2005). All patients received i.m. or i.v MA plus either topical imiquimod or vehicle control. The efficacy was the same for both treatments, but the lesions resolved more rapidly in the imiquimod group, and the residual scarring was less prominent. The adverse events were mild.

In 2006, a randomized, assessor-blind controlled trial in Iran, in a region where *L. tropica* is the predominant parasite species (Firooz et al., 2006) compared the efficacy and safety of i.m. MA with its combination with imiquimod 5% (Aldara®) or with topical placebo. Efficacy evaluated at the end of the treatment period and 4 weeks later showed the addition of 5% imiquimod cream did not improve the response to the treatment with MA.

A prospective, randomized, open trial study in Iran compared the efficacy of topical imiquimod 5% (Aldara®) plus cryotherapy against IL MA. for up to 12 weeks. Follow up was performed until 3 months after the end of treatment. There was no statistical difference in the efficacy between the treatments, but the MA was slightly better than the topical+cryotherapy treatment (Shamsi Meymandi et al., 2011). A randomized, placebo-controlled study conducted in Iraq in 2022 (Dhaher and Hussein, 2022) compared IL sodium stibogluconate plus either topical imiquimod 5% or vehicle. The group receiving imiquimod had higher healing (94 vs. 74%), less scars and less serious scars than the vehicle group.

In summary, topical imiquimod was able to overcome resistance to pentavalent antimonials, the lesions healed faster, and the scars were of better quality than with painful traditional treatments, although in four out of five studies these differences were not statistically significant.

### 6.4 Topical miltefosine

A topical formulation of miltefosine (Miltex) 6% w/v was developed for the treatment of skin-metastasized breast cancer. Two small clinical trials with Miltex for CL, one in Syria (applied twice daily) and a second trial in Colombia (applied once daily for 4 weeks) failed to demonstrate efficacy against CL.

Recently, a liposomal topical formulation of 0.5% miltefosine proved to have a very high efficacy in mice (see Table 3 and Peralta et al., 2021a). Mice however have a different immune system and a much thinner skin than humans, and only the efficacy against *L. amazonensis* was studied (Peralta et al., 2021a). This formulation was studied in a first-in-human, real-life test to treat a patient with resistant CL (Guzman et al., 2023). For two years, the patient had been repeatedly treated with MA, and two treatments with AmB deoxycholate had to be interrupted due to cardiac and renal toxicity. The parasite isolated from the patient was identified as *L. (V) braziliensis.* Miltefosine (0.5 and 1%) was formulated as a liposomal suspension dispersed in a hydrogel. The wound healing was initially good with the topical treatment, but the clinical cure was achieved only with a combination of the topical liposomal miltefosine and two short treatments (6 and 3 days) with intravenous AmB (Guzman et al., 2023).

### 6.5 Topical granulocyte-macrophage colony-stimulating factor

A multicenter project to study a combination of (oral) miltefosine with topical granulocyte-macrophage colony-stimulating factor (GM-CSF) was conducted recently in Brazil to evaluate the efficacy of this combination against *L. braziliensis* and *L. guyanensis*. The placebo-controlled, double-blind clinical trials compared the efficacy of three treatment arms: standard antimonial (Glucantime) therapy, (oral) miltefosine combined with topical GM-CSF, and (oral) miltefosine combined with a topical placebo (vehicle) (Machado et al., 2020). Combining topical GM-CSF with miltefosine did not lead to improved cure rates or a reduction in healing time compared to miltefosine administered with topical placebo. While the median healing time was considerably shorter in the miltefosine arms (60 days) than in the antimonial arm (102 days), the expected synergistic effect from GM-CSF was not observed (Machado et al., 2020; Mendes et al., 2021).

### 6.6 Topical sodium chlorite

A polyacrylate hydrogel containing 0.045% sodium chlorite solution (LeiProtect®) has been studied in patients infected with *L. tropica* and *L. major*. In a two-armed, randomized, double-blinded, phase IIa trial, patients infected with *L. tropica* were treated with bipolar high frequency electrocauterization followed by moist-wound-treatment with vehicle or with hydrogel containing sodium chlorite. The time to wound closure was 76–74 days and 90% of patients were cured, also at 6-months follow up. Observed wound healing times were considerably faster than those previously reported with the standard intralesional antimony treatment in the same endemic environment (Jebran et al., 2014). A three-armed phase IIb, randomized and controlled clinical trial was performed in Afghanistan with *L. tropica- or L. major*-infected CL patients. The treatments consisted of intradermal sodium stibogluconate; high frequency electrocauterization followed by treatment with 0.045% sodium chlorite hydrogel; or 0.045% sodium chlorite in vehicle alone. The lesion closed 3–4 times faster with the physical + topical treatment than with the intradermal injections (Stahl et al., 2014). The efficacy has also been studied in a clinical case of *L. tropica* infection. In this case, only the topical treatment was applied. Complete wound closure was achieved after 8 weeks, at 13 months follow-up the lesions remained cured (Debus et al., 2022).

In summary, although the evidence is weak (only two clinical trials and one clinical case) the topical treatment is very promising, since it was faster and statistically more efficacious than painful traditional treatments. It is however unavailable outside of Germany (see Section 2).

## 7 Conclusion

Cutaneous leishmaniasis continues to be an orphan disease, with treatments that are either very toxic and very painful (pentavalent antimonials, AmB desoxycholate, IL antimonials), or less toxic but very expensive (L-AmB), or not painful but still toxic and unavailable in many endemic countries (oral miltefosine). Physical treatments like thermotherapy are effective but also have limitations.

Inaccessibility of efficacious and less toxic treatments in endemic regions is linked to structural issues such as lack of local manufacturing and donor dependency. Funding cuts in programs like ASCEND further exacerbate this situation. The evidence of efficacy of topical treatments from clinical trials is weak (small or few trials), but a few have very good results. The main problem to increase the solidity of evidence in clinical trials is lack of financing. The pharmaceutical industry is starting to invest in R + D but no new treatments are still on the market, and most of the effort to produce safer, affordable treatment is made by public institutions, universities, and NGOs such as the Drugs for Neglected Diseases Initiative (Kumar et al., 2018). The lack of interest of pharmaceutical companies may change with the evidence of the emergence of the disease in higher-income countries, although it remains to be seen whether this will improve in any way the availability of treatments for patients in low-resources regions. Given the promising results of some clinical studies, in particular those on topical paromomycin, an efficacious topical treatment can surely be available, especially if the continued commitment of publicly or NGO- funded researchers can be supported by an increased interest from pharmaceutical companies (Box 1).

**BOX 1 A brief summary on Post-kala-azar dermal leishmaniasis**

Post–kala-azar dermal leishmaniasis (PKDL) is a chronic skin sequel of visceral leishmaniasis (VL, also called kala-azar). It is observed mainly in areas endemic for *L. donovani* (Zijlstra, 2025). Compared to CL, PKDL is a relatively infrequent complication: the WHO states that from 2012 to 2024, a total of 13,515 cases were reported, with the major burden in East Africa and the Indian subcontinent (World Health Organization, 2024). This low incidence however should be taken as a lowest probable number, since underreporting is likely, due to misdiagnosis (mild lesions, appearance years after VL).

PKDL lesions can appear from 6 months to several years after treatment of VL; they may be hypopigmented macules, papules, nodules, or plaques. Lesions are usually numerous, typically starting on the face and spreading to the trunk and limbs. Some PKDL cases may self-heal over time; in South Asia, lesions usually persist for years without treatment (Ramesh et al., 2015; Kumar et al., 2021).

As PKDL is not a purely local skin disease, representing a systemic persistence of *L.* donovani, systemic drugs are required to fully clear the parasite, prevent relapse, and reduce transmission to sandflies. Topical drugs for PKDL are not established as effective or standard of care, remaining as a minor, investigational niche compared with systemic therapies (Singh-Phulgenda et al., 2024; Moulik et al., 2018; Le Rutte et al., 2019; Mukhopadhyay et al., 2014; WHO, 2012). Miltefosine, liposomal amphotericin B, pentavalent antimonials or combinations of these for 2–4 months are the usual treatments (Singh-Phulgenda et al., 2024; Younis et al., 2023). However, systemic drugs that cure visceral disease may not reach or clear parasites in skin equally; variable skin penetration (and local immune microenvironments) cause treatment failures and relapse. This complicates extrapolating VL regimens to PKDL. In this context, it is important to measure drug concentrations in skin lesions across different drugs and explore higher local exposure (topical formulations, intralesional approaches) or drug delivery systems that increase cutaneous exposure (Wijnant et al., 2024; Palić et al., 2024).
